# PERFECT trial results: Combining neoadjuvant chemoradiotherapy with atezolizumab is feasible in resectable esophageal adenocarcinoma

**DOI:** 10.1111/1759-7714.13972

**Published:** 2021-05-10

**Authors:** Liyi Zhang

**Affiliations:** ^1^ Sun Yat‐sen University Cancer Center; State Key Laboratory of Oncology in South China; Collaborative Innovation Center for Cancer Medicine Guangzhou P. R. China

Esophageal cancer is the seventh most commonly diagnosed cancer and the sixth leading cause of cancer mortality in the world.^1^ It is a heterogeneous disease with high regional variations. The incidence and disability adjusted life years (DLAYs) of esophageal adenocarcinoma (EAC) is rising in western countries, while China contributes the largest number of esophageal squamous‐cell carcinomas (ESCC) cases and burden worldwide.^2^, ^3^ Surgery is the most common treatment for early‐stage esophageal cancer. Previous studies have shown that CROSS (carboplatin, paclitaxel and concurrent radiotherapy)‐based neoadjuvant chemoradiotherapy can extend the median survival of EAC patients with surgery alone from 27.1 months to 43.2 months.^4^ However, approximately 50% of all patients with resectable EAC experience disease progression resulting in death after <5 years of clinical stability.

Esophageal cancer has a unique immune microenvironment,^5^ suggesting that immune checkpoint inhibitors (ICIs) may improve the prognosis of patients with resectable EAC. Indeed, ICIs have been demonstrated to improve the response rate and survival of patients with metastatic gastroesophageal carcinoma,^6^ but the clinical efficacy of ICI in these patients varies greatly. Expression level of programmed death‐ligand 1 (PD‐L1), composition of tumor immune microenvironment and microbiome, and tumor mutational burden are known factors that affect the clinical efficacy of ICI.^6^ Both preclinical and clinical evidence indicate that traditional tumor therapies can change the tumor immune microenvironment of EAC.^7^ For example, taxane‐based chemotherapy can evoke T cell response^8^ and radiotherapy can elevate PD‐L1 expression in EAC.^9^ However, the feasibility and efficacy of neoadjuvant chemoradiotherapy plus ICI for resectable EAC remains to be determined.

In the PERFECT trial recently published in *Clinical Cancer Research*, entitled “Neoadjuvant chemoradiotherapy combined with atezolizumab for resectable esophageal adenocarcinoma: A single arm phase II feasibility trial (PERFECT)”, Ende et al. investigated the feasibility and efficacy of neoadjuvant chemoradiotherapy combined with PD‐L1 inhibitor (atezolizumab) for resectable EAC. A total of 40 patients were enrolled in this study. Among them, 34 (85%) patients received all cycles of atezolizumab and 33 (83%) underwent surgery. Immune‐related adverse events (IRAEs) were observed in six (15%) patients. Reasons for not undergoing surgery were disease progression (*n* = 4; 10%), patient choice (*n* = 2; 5%), and death (*n* = 1; 3%). A total of 10 (25%) patients had achieved complete pathological remission. There was no statistically significant difference in response or survival between the PERFECT and neoadjuvant chemoradiation cohort. The baseline IFNγ‐signature (a six‐gene IFNy‐signature previously established by Ayers et al.^10^) expression of responders was higher than that of nonresponders (*p* = 0.043). The common characteristic of nonresponders receiving treatment is the presence of a large number of cytotoxic lymphocytes with transcriptional signature consistent with the expression of immune checkpoints, or a small number of cytotoxic lymphocytes.

This is the first trial combining CROSS (carboplatin, paclitaxel, and concurrent radiotherapy)‐based neoadjuvant chemoradiotherapy with PD‐L1 inhibitor (atezolizumab) for resectable EAC. Most of the participants (85%) in this study received all planned cycles of atezolizumab suggesting that the treatment regimen is feasible. The neoadjuvant chemotherapy‐related advanced events and postoperative complications reported in the PERFECT trial were basically the same as those reported in the CROSS trial, apart from the immune‐related adverse events associated with PD‐L1 inhibition.^11^ Fortunately, all immune‐related adverse events reported in the PERFECT trial were manageable and did not affect the surgical treatment. Compared with the CROSS trial, the PERFECT trial had a higher pathological complete response rate. In addition, there were more patients who progressed before or during the operation in the PERFECT trial. As the positron emission tomography‐computed tomography (PET‐CT)‐based response evaluation in the CROSS trial was not mandatory, it was very likely that it would to lead to insufficient assessment of disease progression. The pathological response rate, overall survival, and progression‐free survival in the matched cohort of patients treated with carboplatin, paclitaxel, and concurrent radiotherapy was similar to those in the PERFECT trial. Overall, the evidences reported by the PERFECT trial demonstrated that adding atezolizumab to CROSS‐based neoadjuvant chemoradiotherapy does not reduce the number of responders. This observation is completely different from a recent study investigating neoadjuvant atezolizumab plus nabpaclitaxel or carboplatin for non‐small cell lung cancer (NSCLC).^12^ In the NSCLC study, the pathological response rate of patients with neoadjuvant atezolizumab plus chemotherapy was much higher than that of patients with neoadjuvant chemotherapy (33% vs. 5%–8%). However, another important finding in this study was the overall survival and progression‐free survival between the propensity matched cohort and PERFECT were similar, and consistent with two avelumab (a PD‐L1 inhibitor) studies on metastatic gastroesophageal cancer.^13^

In previous studies, the expression of a six‐gene IFNγ‐signature was used to verify the efficacy of pembrolizumab treatment in several cancer types.^10^ Therefore, in the PERFECT trial, the authors attempted to verify whether the expression of IFNγ‐signature could accurately predict which patients will respond to ICI‐based treatment. They found that the responders had higher IFNγ scores than nonresponders. In addition, previous studies found that IFNγ released by T cells could induce the upregulation of PD‐L1 in tumor microenvironment.^14^ Indeed, the baseline combined positivity score (CPS) for PD‐L1 of the majority of responders in the PERFECT trial were ≥ 1–25. Notably, the percentage of patients with baseline CPS ≥10 in the PERFECT trial was relatively higher than that in a recent retrospective analysis of CPS in patients with metastatic gastroesophageal adenocarcinoma (43.6% vs. 15%).^15^ The study of metastatic gastroesophageal adenocarcinoma also performed a paired analysis of primary and metastatic tumors for PD‐L1 expression. The results showed that 36 of the primary tumors were PD‐L1 positive, and 21 (58.3%) were PD‐L1 negative on metastatic biopsy.^15^ However, because the PERFECT trial population did not select PD‐L1 status at baseline, the disease stage and small sample size may affect the CPS. Even so, the evidence from the PERFECT trial still suggests that the IFNγ signature and CPS for PD‐L1 should be further explored as potential biomarkers to predict the response to neoadjuvant ICI therapy in neoadjuvant therapy.

In summary, the PERFECT trial proved that it is feasible to add atezolizumab to CROSS‐based neoadjuvant chemoradiotherapy without affecting the surgical outcomes. Additionally, the immune‐related toxicity of this combination therapeutic strategy was manageable. Compared to the responders of neoadjuvant chemoradiotherapy, the responders in the PERFECT trial had a numerically longer overall survival and progression‐free survival, but the patients treated with neoadjuvant chemoradiation combined with atezolizumab had similar overall survival and progression‐free survival as those who were treated with neoadjuvant chemoradiotherapy alone. Moreover, the six‐gene IFNγ‐signature was associated with the treatment response to neoadjuvant ICI therapy. All these findings lay the foundation for the application of neoadjuvant chemoradiation plus PD‐L1 inhibitor in resectable EAC.

## CONFLICT OF INTEREST

The author declares no competing interests.
